# Managing Paediatric Growth Disorders: Integrating Technology Into a Personalised Approach

**DOI:** 10.4274/jcrpe.galenos.2019.2019.0153

**Published:** 2020-09-02

**Authors:** Jenny Child, Christine Davies, Katie Frost, Eleanor McDermid, Rachel Pidcock, John Weinman, Martin O. Savage

**Affiliations:** 1 The Child Growth Foundation, Sutton Coldfield, UK; 2 University Hospital of Wales, Noah’s Ark Children’s Hospital for Wales, Children’s Hospital, Cardiff, UK; 3 The Straw Barn, Upton End Farm Business Park, Bedfordshire, UK; 4 Springer Healthcare IME, The Campus, London, UK; 5 King’s College London, Institute of Pharmaceutical Science, London, UK; 6 Barts and the London School of Medicine and Dentistry, William Harvey Research Institute, Centre of Endocrinology, London, UK

**Keywords:** Growth, growth hormone, adherence, motivational interviewing, eHealth

## Abstract

Long-term growth management can be challenging for patients, families and healthcare professionals (HCP). Personalised optimal responses to growth hormone (GH) therapy depend on the creation of a good working relationship between the patient and family and the HCPs responsible for care. Current unmet needs in growth management will be discussed, focusing on the likelihood of a poor growth response and its identification and management with emphasis on the importance of good adherence to GH therapy. Digital tools are now available to record injections and communicate accurate adherence data to the HCP and patient. Psychological barriers to good adherence will be covered, with techniques identified to change behaviour and improve outcome. Motivational interviewing is a valuable skill in this respect and should be taught to both medical and nursing HCPs to enhance the quality of the relationship with the patient and family. Key messages are, firstly, the importance of personalised care with the HCP using acquired psychological skills to prevent and manage poor adherence. Secondly, a human-eHealth partnership is necessary to maximise the benefit of new digital tools to aid in successful growth management.

**What is already known on this topic?**

There have been few articles specifically linking the human component of growth management, i.e. specialist and nurse interaction with the patient, psychological support and training of healthcare professionals in motivational interviewing together with digital innovations such as electronic monitoring of growth hormone (GH) injections. Both the human and digital components are recognised to contribute to GH adherence, but it is the necessity of their partnership that we emphasize.

**What this study adds?**

A review on the holistic approach to personalised growth management by multi-disciplinary professionals, but stressing the key importance of the human and technical partnership. Contributions are also provided by a professional coach who is an expert in motivational interviewing and personnel from the UK patient support group, the Child Growth Foundation.

## Introduction

The management of paediatric growth disorders presents a multidisciplinary challenge to healthcare professionals (HCPs) responsible for affected patient care. Several medical HCPs may be involved, including the primary care physician who identifies the initial growth problem, the family general practitioner who refers the child for hospital investigation, the hospital-based paediatrician who sees the child at the initial consultation and the specialist paediatric endocrinologist to whom the child is then referred for an expert opinion and further management. In addition, in many hospital paediatric endocrinology units, the developing role of the paediatric endocrinology nurse specialist has directly improved the quality of liaison with the family and contributes to the care of the child through the addition of a skilled HCP to the management team. Pharmacists, biochemists, psychologists, patient support groups and personnel from the pharmaceutical industry also make important contributions to the three key phases of growth management; namely identification of the initial short stature, investigation and diagnosis of the cause, and treatment with hormone therapy, where indicated, all of which implies a long-term commitment to a potentially invasive therapy (1). Early diagnosis and early initiation of growth hormone (GH) therapy is associated with improved long-term height gain (1,2).

The pressures experienced by the patient and family to successfully engage in such a diagnostic and therapeutic journey are also challenging. There are key facts about the nature and implications of the diagnosis to understand and process, including the emotional commitment for therapy to be successful and produce normal growth and adult height. In addition, maintenance of a therapeutic regimen designed to bring long-term improvement, rather than short-term benefit, requires engagement and maturity.

These aspects of short stature management will be discussed in this article. A further component of care, which has emerged in recent years, are the electronic tools to aid therapy and adherence. These tools will also be addressed with emphasis on the importance of the human-eHealth partnership, which is necessary to make patient care optimally beneficial.

We will discuss the challenges encountered by the patient and family through the experience of staff of the UK Child Growth Foundation (CGF), a patient support charity which advises families of patients with short stature. Current unmet medical needs of growth management will also be discussed (1) followed by a description of the psychological basis and management of poor adherence to GH treatment regimens (3). eHealth innovations will be covered, followed by the importance of HCP training in relation to acquisition of motivational skills for improved recognition and intervention in poor adherence situations. Finally, the emerging role of the paediatric endocrinology specialist nurse will be summarised, with conclusions highlighting the rationale for joint human-eHealth collaboration to achieve optimal personalised management of the short child.

## Current Unmet Needs in the Management of Short Stature

Early recognition of pathological short stature, as opposed to variants of normal height, remains a challenge, particularly in the UK, where routine height surveillance has been reduced to two measurements at primary school and secondary school entry points (4). The age of diagnosis of disorders of abnormal growth, such as coeliac disease and Turner syndrome, is significantly later than in other countries such as the Netherlands and Finland (5), where investment in primary care identification of growth disorders has resulted in earlier diagnoses (6).

Historically, a high proportion of children, treated with GH therapy for a variety of growth disorders, have not demonstrated a satisfactory degree of catch-up growth during the first year of therapy (7). A number of reasons may underlie this, including incorrect diagnosis, incorrect dose of GH at initiation of therapy and inadequate attention to factors predicting individual growth responses (8). The correct management of poor response to GH remains a priority in such patients (9). However, it is the presence of poor adherence to the GH treatment regimen which has emerged as a key factor, either alone or in combination with other elements that have an impact on growth response (10,11). This issue of non-adherence will be discussed in detail below.

## Digital Advances in the Management of Growth Disorders

Digital health, defined as the use of information and communication technologies for health, is becoming a reality in clinical practice and medical education and has made a significant impact in the day-to-day management of diabetes mellitus in children (12). Its application to the treatment of growth disorders is more challenging because therapy is geared to long-term responses and benefits, rather than short-term metabolic control. However, one area where digital technology has been effective is in the electronic monitoring of GH injections (13,14). The use of an electromechanical auto-injector, which records every injection that is given and communicates the data both to the patient and the HCP, is a major advance (15). It is known that self-reporting of adherence tends to be inaccurate and to report artificially high values, compared with digital recording of injections (16). The difference between reported and recorded accuracy, using the electronic device, is significant.

In a large international study of GH therapy using electronic recording, adherence was shown to be good during the first year of treatment, but gradually decreased to approximately 60% after five years (13). These data give two key messages, first that accurately measured adherence decreases over time and secondly that intervention by the HCP is indicated to prevent and correct this trend. The injection device can also demonstrate suboptimal adherence which may not be obvious from auxological measurements.

## Psychological Factors Predisposing to Non-adherence: The Human-digital Model of Collaborative Care

Adherence or compliance can be defined as the extent to which the patient follows a prescribed therapeutic regimen, and in the case of GH, the extent to which daily GH treatment is taken. There are three phases in understanding the way adherence develops. First, there is the uptake stage, which describes the way in which the patient begins to accept the treatment and indeed whether they actually start to take the treatment. It is known that 10% to 15% of patients never start taking the treatments they are prescribed (3). This is known as primary non-adherence. The second phase, which is really critical for long-term progress, is the way in which the patient, or the family, incorporates the treatment into the habitual pattern of daily life. The last phase describes how long the patient stays with the treatment.

It is known that patients may give up after months or years of treatment and there is evidence for a wide range of adherence to GH therapy (3,11). Overall, there are figures of up to 50%, 60%, or even 70% of patients not taking GH treatment in a regular and useful way, with a clear relationship demonstrated between non-adherence and not achieving linear growth targets (10).

Given that GH therapy is evidence-based, the question is why are patients not adherent? Older explanations were essentially based around the idea that people did not follow treatment because they did not understand or remember what they had to do (17). This was often taken to be a symptom of poor communication in healthcare, so interventions were designed to improve communication and patient understanding and the ability to remember and plan treatment. This, unfortunately, is only a small part of the answer.

## Intentional and Non-intentional Non-adherence

**Figure 1:**
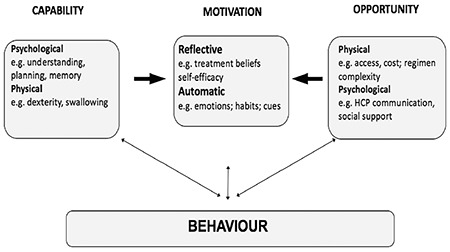
The COM-B model: A new approach to classifying adherence to diseases (adapted from reference: 18) HCP: healthcare professionals

It is now clear that there are different categories and certainly different causes of non-adherence. Two distinct types are recognised, known as intentional and unintentional non-adherence, which have very different drivers, or different origins. The reasons for the two different categories can be summarised in terms of what is known as the COM-B model (Figure 1) (18). In the COM-B acronym, C stands for capability, O for opportunity and M for motivation. In intentional non-adherence many patients know what they have got to do, ie it is not a question of misunderstanding or not remembering, but they are reluctant to adhere, because either the treatment does not make sense to them, or they have worries or concerns about it. In unintentional non-adherence some of the older factors can be responsible such as poor communication, poor experience or satisfaction with the organisational challenges of doing something regularly on a daily basis. There may also be other barriers outside the individual, such as financial or practical constraints.

## The COM-B Model

If this is mapped onto the COM-B model (Figure 1) we can see that under **Capability**, there is a range of factors, such as psychological difficulties; eg, people not remembering or not being able to plan. There are also some physical capability issues, eg, not being able to administer the treatment in a way that is effective. Under **Opportunity**, there are physical factors such as getting access or having barriers to treatment, which lie outside the patient, together with psychological barriers, such as poor support and communication from people close to the patient. However, the really important factors for many patients, particularly related to intentional non-adherence, are the **Motivational** influences, such as negative or mistaken beliefs about their condition and their treatment.

## Human and Digital Interventions

Accepting this variety of factors, it is not surprising there is a range of ways that we have of working with families and patients, to improve their adherence. These can involve both human and digital interventions. Two available strategies are equally important. It is fundamental to use the direct experience in the healthcare situation, ie the consultation, to understand the patient’s issues and perspectives and to anticipate factors around non-adherence which can be managed. Going beyond that, there is a range of digital and personalised interventions available; for example, an initial brief screening questionnaire to identify the particular problems each patient and family may be experiencing. Then, following that, interventions can be developed which are tailored to each patient.

In terms of the consultation, a structure is recommended for each family to analyse their understanding of the primary short stature condition and the treatment regimen they are being asked to follow. It is important to make sure that they have a clear rationale for the need for treatment and for daily injections. A recent study in adults with GH deficiency showed that non-adherence was related to lack of understanding of the primary disorder, which can be improved through focused education (19). A practical plan needs to be agreed for how, where and when the GH injections are given to ensure that treatment is administered more regularly.

More generally, factors which cause adherence problems for each individual need to be identified. At the beginning and during treatment, brief screening questionnaires can be used to identify relevant personal issues. Information from the screening questionnaires can be used to start a personalised conversation to understand what is going wrong. From there, basic behaviour change approaches, such as motivational interviewing by HCPs, can attempt to target individual factors.

Beyond the consultation, many other digital approaches are available which patients and parents can access on a daily basis. These could be personalised web-based tools, mobile phone applications, daily text messaging or interactive programmes which address particular issues.

## Training of Medical and Nursing HCPs in Motivational Interviewing to Address Family Interactions Related to Growth Management

The main role of a professional coach in the healthcare environment is to support HCPs in learning how to help patients to make healthy choices and decisions in their lives. This can be challenging because patients can struggle to make such choices, particularly when emotional barriers block the logical courses of action. A number of questions can be asked. How can HCPs really influence the behaviour of patients and families, particularly when they have decided they do not want to change? Why can some patients move forward when others are resistant to making progress?

These questions and observations have led to the exploration of motivational interviewing practised by HCPs which can be applied in the clinical scenario of outpatient consultations to help patients with adherence to GH therapy. It is proposed that motivational interviewing skills can motivate patients and families to overcome the practical and emotional barriers related to therapy.

## Motivational Interviewing

Motivational Interviewing, which is based on the work of Miller and Rollnick (20), is a collaborative conversation style which aims to strengthen a person’s motivation and commitment to change. It is a structured, person-centred approach which helps patients and families to resource their own inner motivation to be translated into improving adherence to GH therapy. Motivational interviewing is a skill which needs to be taught and thus learnt by both medical and nursing HCPs.

Examples of the benefits of motivational interviewing can be taken from experience in making healthy life choices, such as giving up smoking, reducing alcohol intake or eating in a healthier way. When considering these choices, reaction to the individual can be unhelpful, such as not listening or negatively encouraging regressive behaviour. By contrast, a helpful response to the same life choices would consist of positive reactions such as genuine empathetic listening and exploration of the individual’s feelings without judgement. This behaviour typifies the spirit of motivational interviewing.

The principles of motivational interviewing are collaboration, acceptance and compassion. Collaboration is very important because partnership on an equal level with the patient is a key aim. Acceptance leads to better understanding of the decisions and choices that patients and families are making without judgement. These choices are accepted and the HCP responds with guidance. Compassion is a further component that is combined with evocation, which means drawing out a patient’s inner motivation and commitment, and building on this to effect change.

## The OARS Model

Core skills in motivational interviewing can be discussed under the acronym OARS, which stands for Open questions, Affirmations, Reflective listening and Summarising. The conversation can be structured by following these headings. Open questions such as what, how and why will open conversations and evoke dialogue. Other examples would be ‘what are your hopes for your consultation today?’ and ‘I am curious to learn how you have been getting on with your injections?’ These questions can be prefaced by saying ‘help me understand …’ and the conversation can develop by inviting the patient or family to talk about what is on their mind, what are their needs and their priorities. Affirmations are about helping patients to recognise their own strengths and positive beliefs that are going to help them to adhere to GH therapy. Examples could be to say to a patient ‘I can see it took courage for you to try this out today’ or to a parent ‘your creative ideas around this are very helpful’. Reflective listening consists of not only listening and reflecting back what is said, it also helps in verbalising the thinking and feelings that lie underneath, showing a depth of empathy that leads to further conversations. The last skill here is summarising, which serves the useful purpose of wrapping up conversations and can be started by saying ‘let me see if I have got this right, you are feeling this on one hand and perhaps feeling this on the other?’

## Challenges with Adherence from the Patient and Family Perspectives

When patients and families are asked about the difficulties they face related to management of short stature, a wide range of opinions and comments are given. The UK CGF (https: www.childgrowthfoundation.org) is a non-profit patient support group, which was originally founded as a charity in 1977 (UK Registered Charity number 1172807). The CGF receives many requests for information and support and delivers management advice on a wide range of growth disorders.

In relation to adherence to GH therapy, the CGF reports that in the consultation setting some HCPs do not have sufficient time or experience of GH treatment which results in them giving conflicting advice to families. Insufficient knowledge of the primary growth disorder results in communication of inadequate or incorrect information. In particular, the patient may not realise how effective and worthwhile long-term therapy with GH can be. Insufficient education of the patient by the HCP can result in the family seeking alternative advice on the internet and thus receiving more confusing, incorrect and worrying messages. More accurate information needs to be available regarding the benefits of GH therapy with advantages outside growth being emphasised, such as improved general health and self-esteem (19). Accurate information regarding GH injection devices needs to be given with the choice of the most suitable injection device made by the family before the initiation of therapy. Size, comfort and storage requirements should also be considered, together with family dynamics and travel.

## Patient Choice of GH Brand and Injection Device

**Figure 2:**
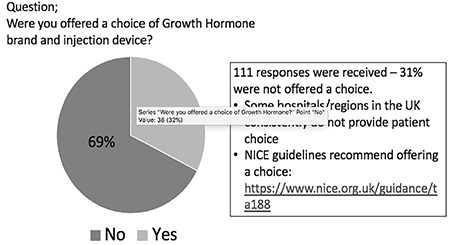
Results of a patient-directed survey on choice of growth hormone brand and injection device

The concept of patient choice is an organisational decision which is not universally adopted in the framework of growth consultations. Ideally however, the patient and family should be offered the choice of GH brand and injection device and this has been demonstrated to increase the likelihood of good adherence (21,22). In 2019 the CGF conducted an online survey amongst its members about initiation of GH therapy (Figure 2). One hundred and eleven responses were received, mostly from patients with GH deficiency, multiple pituitary hormone deficiencies, Silver Russell syndrome, small for gestational age and intrauterine growth retardation. The two most relevant questions were, ‘Were you offered a choice of GH brand and device?’ and ‘How often does your child miss a GH dose?’ Out of 111 responses, 31% of patients were not offered a choice of GH brand or injection device, demonstrating that within the UK, patient choice remains very inconsistent. Guidelines for England and Wales, regarding GH treatment, https: www.nice.org.uk/guidance/ta188/chapter/1-Guidance are not being followed. The survey indicated that 58% of patients never missed a GH dose, with lower values of 30% of non-GH deficient cases compared with 78% in multiple pituitary hormone deficiency cases.

## Family Logistical Barriers to Good Adherence

From many years’ experience of handling requests for information and from managing the CGF Facebook page, the CGF reports topics, which are frequently repeated, related to barriers to good GH adherence. The first of these is logistical barriers. A daily subcutaneous injection should become part of the family’s routine, provided the routine is not disturbed. However when changes do occur, such as a play-date, a school trip, a sleep-over, a camping trip with refrigeration necessary or particularly when the child’s care is shared between parents in different locations or with grandparents, the first casualty is the GH injection. As the effect of missing one or several GH injections is not immediately apparent, the long-term objective of regular therapy tends to be forgotten leading to chronic poor adherence. Another practical aspect is the maintenance of regular GH supplies, which may not occur if a family waits to order a new supply at the last minute.

## Emotional Barriers to Good Adherence

Children receive GH treatment because they have a long-term health condition but may develop a needle phobia with a fear of the pain of the injection combined sometimes with the noise of the injection device. A vicious cycle of events can develop and escalate in importance, predictably leading to missed injections. The anticipation of the injection and then its attempted administration can be very stressful. In the longer-term, a child might start to feel different to their peers, especially around friends, of whom not many will be having daily injections. Bullying and exclusion of the patient can occur. Peer pressure increases during adolescence, when additional stresses, such as exams, provide further opportunities to miss GH injections and for poor adherence to become habitual.

## Benefits of Peer Support

Availability of communication with other patients having similar experiences can be very supportive and can significantly reduce stress and the sense of isolation. Peer support organisations such as the CGF can support and advise their own patients and the HCPs who are responsible for them. Many host social media groups, providing a 24/7 online community for chats, questions, discussions and mutual support. The CGF holds an annual convention, but with e-technology, geographical boundaries have diminished and Facebook groups, educational websites, mobile phone applications and helplines can all contribute to enhanced patient and family support.

## Contributions of the Paediatric Endocrinologist Specialist Nurse to Practical GH Management

The roles of the paediatric endocrinology specialist nurse have developed at different rates in different countries. In the USA, UK, Canada, Australia and Scandinavia this nursing speciality has grown, with funding now established for positions in most university paediatric endocrinology departments (23). In other countries paediatric endocrinology nursing is much less developed. We will discuss roles and responsibilities related to short stature management and specifically GH adherence.

Paediatric endocrine specialist nurses are uniquely positioned to offer a high-valued support network to HCPs, patients and their families, by being the regular first point of contact at consultation visits. Relationships, incorporating the whole family, are established and built on trust, specialised knowledge and expertise that is pivotal for families when starting GH therapy. Involvement in the initiation of GH treatment is key to establishing a fruitful relationship with the patient. ‘Ideal’ and ‘worst-case’ scenarios regarding initiation of GH therapy are shown in Table 1. If possible, meeting the family before the medical consultation can be very beneficial. Obtaining knowledge of the medical history and whether the family has studied the diagnosis on the internet can also be very valuable.

Communication skills are important and as discussed above, training in motivational interviewing can play an essential role in the specialist nurse becoming an effective member of the growth management team and contributing to optimal GH adherence. Organising the patient’s choice of GH brand and injection device is a further responsibility and needs to be based on specialist knowledge of the different GH devices.

Education in injection technique will logically lead to the establishment of a network of regular contacts and availability for the patient and family. Contact and support by phone and internet have become inherent in the nurse specialist’s responsibilities. In terms of adherence, the use of electronic monitoring of injections with feed-back to the nurse and endocrinologist allows adherence to be examined, so that a human-eHealth partnership develops to support the family. At consultation visits, it is logically the nurse specialist who can take the lead in non-judgemental interviewing to investigate actual or potential non-adherence.

In the long-term, the paediatric endocrinology specialist nurse maintains support and positive relationships with the family and the patient. Everyone needs to continue to work together, ensuring encouragement and a combined committed goal of optimal response to GH therapy. Finally, by using a personalised approach, technology can be positively integrated into care and assist adherence and optimise outcomes.

## Conclusion

The successful management of paediatric growth disorders, involving GH therapy, can be judged by the achievement of catch-up growth, followed by growth within the normal centile lines leading to an adult height within the genetic target of the family. Relatively few cases achieve this ideal triad and a combination of personalised input by medical and nursing HCPs and the use of technological tools can improve the chances of success. Understanding the personal psychological barriers to good GH adherence in each patient can be combined with the use of an electronic GH injection recorder to monitor and communicate accurate adherence data. Motivational interviewing and a non-judgemental approach are also beneficial. This human-eHealth partnership gives synergistic advantages and improves the likelihood of a clinically beneficial long-term growth outcome.

## Figures and Tables

**Table 1:**
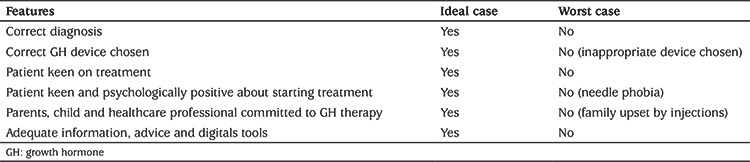
Ideal and worst-case scenarios when starting growth hormone therapy from the point of view of a paediatric endocrinology nurse specialist
